# Cancer and Autoimmune Diseases: A Tale of Two Immunological Opposites?

**DOI:** 10.3389/fimmu.2022.821598

**Published:** 2022-01-25

**Authors:** Zeev Elkoshi

**Affiliations:** Research and Development Department, Taro Pharmaceutical Industries Ltd, Haifa, Israel

**Keywords:** cancer, autoimmune diseases, reactive oxygen species, standardized incidence ratio, hazard ratio, regulatory T cells, inflammation, major histocompability complex

## Abstract

The present article compares, side-by-side, cancer and autoimmune diseases in terms of innate and adaptive immune cells involvement, MHC Class I and Class II expression, TGFβ effect, immune modulating drugs effect and the effect of reactive oxygen species. The change in the inflammatory immune reaction during the progress of cancer and the effect of this change on the comorbidity of autoimmune diseases and cancer are discussed. The similar inflammatory properties of autoimmune diseases and early cancer, and the contrasting inflammatory properties of autoimmune diseases and advanced cancer elucidate the increased incidence of many types of cancer in patients with pre-existing autoimmune diseases and the decreased cancer-specific mortality of these patients. Stage-dependent effects of reactive oxygen-species on tumor proliferation are an additional probable cause for these epidemiological observations. The relationship: {standardized incidence ratio (SIR)} > {cancer-specific hazard ratio (HR)} for cancer patients with a history of autoimmune diseases is substantiated and rationalized.

## Introduction

Earlier work proposed a classification of chronic diseases into pro- and anti-inflammatory diseases based on the extent of regulatory T cells (Treg) activity observed (“high Treg” or “low Treg”) (1). This classification was found useful (a) in understanding the efficacy of drugs for the treatment of different chronic diseases, (b) in explaining the association of specific chronic diseases with specific pathogens, and (c) in interpreting the effects of different pathogens on cancer and autoimmune diseases (1–3).

The present work expands on the immunological properties of cancer and autoimmune diseases (AIDs) in relation to the binary classification proposed. During its course of growth, cancer progress from a pro-inflammatory, “low Treg” disease, to an anti-inflammatory, “high Treg” disease. This change is observed not only within the tumor microenvironment (TME) but also in the systemic circulation [Ref. 2 and references therein]. On the other hand, many autoimmune diseases are characterized by a decreased Tregs function or frequency (4), and may be regarded as proper pro-inflammatory “low Treg” chronic diseases. In addition, as will be described later, reactive oxygen species (ROS) exert opposite effects on the promotion of AIDs and *advanced* cancer. For these reasons, AIDs and advanced cancer can be considered immunological opposites. In contrast, AIDs and *early* cancer are triggered and promoted by a similar anti-inflammatory environment. As will be portrayed later, perspicacity of these time-related relationships helps in explaining the increased risk of cancer development in patients with AID history, a commonly reported observation which was referred to in the literature as “a paradox” (5). Noticing these unique relationships also helps in elucidating the longer survival of cancer patients with pre-existing AIDs compared to cancer patients without documented AIDs.

In the next two chapters, immune reactions to cancer and AIDs, and the effect of ROS on these immune reactions, including the roles of the major histocompatibility complex Class I (MHC-I) and Class II (MHC-II) and transforming growth factor β (TGFβ) will be reviewed and compared. The comorbidity of cancer and autoimmune diseases and the effect of ROS on this comorbidity will then be discussed.

## Cancer

### The Involvement of T Cells in Cancer

CD8+ T cells attack cancer cells directly, and are of primary importance among several anti-cancer mechanisms reported in animals and man. By recognizing peptide-MHC-I complexes, CT8+ T cells identify cancer cells and destroy them through the release of perforin and the activation of the FAS apoptosis pathway (6).

Tregs suppress the function of CD8+ T cells (7, 8) and thus promote cancer. In addition, Tregs inhibit the proliferation of memory T cells (9) and of effector T cells such as Th1 (10) and Th2 (11). Th1 cytokine IL-2 demonstrated an anti-tumor activity in experimental models (12). A recombinant form of IL-2 is approved in several countries for the treatment of malignant melanoma and renal cell carcinoma. However another Th1 cytokine, IFNγ, exhibits pro- and anti-tumor effects (13).

In contrast to Th1 mediated immunity which is in general anti-cancer, Th2 mediated immunity present pro- and anti-tumor effects (14).

Th17 cells may display a pro- or anti-tumor role within the TME (15). Their frequency in the circulation of cancer patients increases compared to that in healthy controls. This was reported in many cancers, including renal cell carcinoma (16), ovarian cancer (17), breast cancer (18), cervical cancer (19, 20), endometrial cancer (21), hepatocellular carcinoma (22), and oral squamous cell carcinoma (23). However, low values of Th17 cells in the circulation of cancer patients have been reported in pancreatic cancer (24) and lung cancer (25). Th17 cells accumulate in the TME of cancers such as gastric cancer (26), breast cancer (18) and prostate cancer (27).

Regulatory T cells expansion in the peripheral blood of cancer-patients has been documented in ovarian cancer (17), hepatocellular carcinoma (22), oral squamous cell carcinoma (23), pancreatic cancer (24), gastric cancer (26), renal cell carcinoma (16) and cervical cancer (19, 20). High frequencies of Treg cells in the tumor microenvironment (compared to non-tumor tissues) were observed in breast cancer TME (18) and lung cancer TME (25). A correlation between Treg cells frequency and cancer progression was observed in breast cancer (18), lung cancer (25), hepatocellular carcinoma (22), oral squamous cell carcinoma (23), and gastric cancer (26),

Collectively, accumulation of Treg and Th17 cells in the tumor microenvironment along with increased levels of both cells in blood are observed in many cancers.

The effect of Th17 cells on cancer is usually limited. Inhibition of IL-23, a key cytokine for Th17 maintenance and expansion, by IL-23 inhibitor risankizumab, does not increase long-term cancer risk (28). Similarly, long-term treatment of psoriasis, psoriatic arthritis and ankylosing spondylitis patients with IL-17A inhibitor secukinumab, demonstrated a long-term low risk of malignancy (29). A pooled analysis of other long-term studies with secukinumab presented similar results (30). Another IL-17A inhibitor, ixekizumab, also demonstrated a long-term low risk of cancer (31). However other studies with these and other IL-23 inhibitors display conflicting results with pro-tumor or anti-tumor effects (32). It may be concluded that Th17 cells do not present significant anti-cancer or pro-cancer activities.

### The Involvement of Neutrophils in Cancer

Tumor associated neutrophils (TANs) display plasticity in the TME. In the absence of TGFβ they attain a pro-inflammatory and anticancer mode of action (N1 phenotype) while in the presence of TGFβ they present an anti-inflammatory and pro-cancer response (N2 phenotype). The N1 phenotype which produces high levels of TNFα, NO and H_2_O_2_, prevails in the TME of early tumors when the level of TGFβ is low. The N2 phenotype predominates at later stages when TGFβ accumulates (33). Circulating neutrophils in tumor-bearing mice display two sub-populations classified by their densities in peripheral blood mononuclear cells (PBMC) fraction, after density gradient centrifugation of whole blood. High density neutrophils (HDN) prevail in tumor-free mice while low density neutrophils (LDN) are the dominant neutrophils in tumor-bearing mice. HDN were cytotoxic towards cancer cells in culture, whereas LDN were innocuous. Similar to TANs, high density neutrophils transform into the low density type in a TGFβ-dependent manner (33). It is not surprising therefore that circulating blood of patients with advanced-stage lung cancer was reported to be enriched with LDN, relative to early-stage patients, or healthy controls (34).

### The Involvement of Macrophages in Cancer

Macrophages can be classified as pro-inflammatory macrophages (M1) or anti-inflammatory macrophages (M2). M1 macrophages promote inflammation by the secretion of inflammatory cytokines such as TNFα, IL-1α, IL-1β, IL-6, IL-12, IL-18, and IL-23, and by the generation of ROS and reactive nitrogen species. They also express high levels of MHC that allow the activation of the adaptive immune arm. M2 macrophages secrete high levels of IL-10, PGE2 and TGFβ, cytokines that play an anti-inflammatory role. M2 macrophages express high levels of arginase-1, mannose receptor, and low levels of MHC class II complex (35).

Tumor associated macrophages (TAMs) include both phenotypes, M1 and M2. It has been proposed that macrophages display a pro-inflammatory antitumor effect (M1 phenotype) in early cancer and an anti-inflammatory pro-tumor effect (M2 phenotype) in established cancer (36).

### The Effects of ROS on Cancer

The tumor microenvironment is rich in reactive oxygen species. As the tumor develops, ROS accumulate in the TME of many solid cancers, as a result of intensive production by tumor cells mitochondria and nicotinamide adenine dinucleotide phosphate (NADPH) oxidase. In addition, ROS are released into the TME by cancer-associated fibroblasts (CAFs), TAMs, and myeloid-derived suppressor cells (MDSCs) (37). The effects of ROS on cancer proliferation are complex. They are partly mediated by the interaction of ROS with Tregs, but other pathways are possible. These interactions are presented below.

#### ROS Are Required for Tregs Function

When mitochondrial oxidative phosphorylation in Tregs is impaired by the ablation of mitochondrial respiratory chain complex III in mice, the suppressive capacity of these Treg cells is lost without altering Treg cell proliferation and survival (38).

#### ROS and Tregs Mutually Induce Each Other’s Activity

It has been demonstrated *in vitro* and *in vivo* that ROS generated by macrophages NADPH oxidase complex induce Treg frequency and function (39). In addition it was shown in psoriasis rat model that ROS prevent imiquimod-induced psoriatic dermatitis by enhancing Tregs function (40). On the other hand, TGFβ excreted by Treg cells induced the generation of ROS by NADPH oxidase (41, 42). In accordance with these observations, Treg-induced immunosuppression in the tumor microenvironment is mediated by Tregs-generated ROS (43).

#### ROS Induce MHC-I Expression in Cancer Cells (Which Promote the Anti-Cancer Effect of CD8+ T Cells)

MHC-I expression in tumor cells has been shown to increase following oxidative stress (44, 45). This increased expression of MHC-I facilitates the identification of cancer cells by CD8+ T cells and boosts tumor attack by these T cells.

#### Reduction in ROS Production by Dendritic Cells Hampers Their MHC-I Machinery and Impairs Their Role in Anti-Cancer Immunity

Mitochondrial reactive oxygen species have a role in cross-presentation of MHC-I antigens by plasmacytoid dendritic cells. Reduction in mitochondrial generation of reactive oxygen species by plasmacytoid dendritic cells resulted in a substantial decrease in the ability of these cells to induce a CD8+ T cell reaction following cross-presentation (46). This behavior was also reported in normal dendritic cells, where NADPH oxidase isoform NOX2 (an enzyme with a prominent role in mitochondrial reactive oxygen species generation) was found essential for an efficient antigen cross-presentation to CD8+ T cells. Lack of NOX2 in DCs resulted in impaired cross-presentation (47). Dendritic cells from chronic granulomatous disease patients with an impaired catalytic subunit of NOX2 have shown defective cross-presentation of soluble antigens to CD8+ T cells (48). Since dendritic cells are major players in the control of cancer by adaptive immunity (49), a reduction in ROS production by dendritic cells is expected to promote cancer.

#### Moderately-High ROS Levels Promote Solid Cancers While Low ROS Levels Are Cytostatic and Excessive ROS Levels Are Cytotoxic

As discussed above, ROS effect on tumor growth may be mediated by Tregs. However other routes for the effect of ROS on malignancy are possible. At the pre-cancerous stage, ROS may drive cancer initiation by inducing oxidative damage and base pair substitution mutations in tumor suppressor genes such as TP53 (50). As the tumor grows, the increased level of ROS in the TME induces tumor proliferation by enhancing Tregs activity. In addition ROS activate several canonical pathways involved in tumor propagation such as the NF-κB pathway, the MAPK pathway (including ERK1/2, JNK, MAPK-11 and MAPK1) and the PI3K/PTEN pathway (37). Reactive oxygen species also support the development of tumor metastases by driving epithelial–mesenchymal transition (EMT) (48, 51, 52) and by anoikis inhibition (anoikis is a type of programmed cell death induced by cell detachment from extracellular matrix) (53).

These and other pro-cancerous effects of increased ROS levels drive tumor progression. However, very high levels of oxidative agents may result in tumor tissue damage. Excessive ROS levels induce cell death by apoptosis, necrosis and ferroptosis (54). Hence, a moderately-high level of ROS within the TME would be optimal for tumor growth and sustainability (55–57). A tight control on ROS balance is therefore required for tumor progression. It may be stated that moderately-high intra- and extra-cellular ROS levels promotes solid cancer propagation, metastases and angiogenesis, whereas very high levels induce cancer cell death and low levels are cytostatic.

### The Involvement of MHC-I in Cancer

#### MHC-I Induces the Suppressive Function of Tregs Which Promotes Cancer

Using a mouse model, Mu et al. have shown that MHC-I transcription is enhanced by the transcription factor FoxP3 in T cells. This resulted in a higher expression of MHC-I in CD4(+)CD25(+) regulatory T cells than in conventional CD4(+)CD25(-) T cells. The authors also found that MHC-I expression by Tregs contributes to their regulatory function (58). High suppressive Treg activity drives advanced cancer and metastases (2). Mu et al. mention a study by Joetham et al. reporting that CD8+ T cells interaction with MHC-I on Tregs is required for Tregs activation (59). In relation to this observation, TCRs expressed in peripheral Treg cells can recognize foreign antigens with high affinity. Tregs induce antigen-specific suppression mainly by Treg-DCs interaction. A mechanism by which DCs present antigens to Treg cells as part of MHC-II, by means of Treg TCR, has been proposed. This process eventually results in the generation of antigen-specific tolerogenic DCs (60). One may speculate that antigens presented by DCs to Tregs may further bind to MHC-I to form protein-MHC-I complex. This complex may help CD8+ T cells in recognizing Tregs (the way CD8+ T cells recognize cancer cells), before they activate them by a direct contact.

#### TGFβ Suppresses MHC-I Expression

The suppression of MHC-I expression by TGF-β1 has been demonstrated in the TGFβ1 null mouse where elevated mRNA levels of MHC-I (and MHC-II) were detected compared to normal or TGFβ1 heterozygous littermates (61). Incubation of two human uveal melanoma cell lines in the presence of TGF-β generated more than 50% decrease in MHC-I antigen expression (62).

#### Tumors Escape Immune Control Through the Loss of MHC-I Antigen Presentation Machinery

As explained above, peptide-MHC-I complexes guide tumor attacks by CD8+ T cells. Many solid tumors demonstrate decreased expression of MHC-I antigen presentation (mediated through different pathways), and thus evade cancer immune control by CT8+ T cells (63).

### The Involvement of MHC-II in Cancer

MHC-II-mediated antigen presentation to CD4+ T cells supports immune response by both T helper cells and CD8+ T cells [CD4+ T cells are required for CD8+ priming and function (64)]. Even though MHC-II is expressed mainly by professional antigen presenting cells (macrophages, dendritic cells, and B cells) and by thymic epithelial cells, other cells may express MHC-II, including cancer cells. Indeed, in addition to changes in MHC-I expression by cancer cells, changes in the expression of MHC-II by cancer cells are also observed (65). The frequencies of MHC-II alleles in cancer patients are different from these observed in healthy subjects. These differences, which depend on the type of cancer, were found to correlate with the risk of developing the malignancy and with the response to treatment (66). It is tempting to believe that CD+4 T cells (and MHC-II expression) are important for anti-tumor immunity. In line with this, the response to PD-1 blockage in a synergistic murine model of melanoma required CD4+ T cells in addition to CD8+ T cells, and a costimulation by dendritic cells and macrophages (67).

#### Tregs Deplete MHC-II From Dendritic Cells

Akkaya et al. have shown in a mouse model that one of the mechanisms by which regulatory T cells induce immune suppression is by the depletion of peptide-MHC-II complex from DCs (68). Since advanced cancer is a “high Treg” disease, this regulatory mode of action is expected to promote immune escape at advanced cancer stages.

#### TGFβ Reduces MHC-II Expression in Macrophages

Delvig et al. demonstrated that by reducing MHC-II expression on macrophages, TGFβ blocked antigen presentation of two T cell epitopes. In addition, TGFβ reduced the constitutive expression of MHC-II transactivator (CIITA), invariant chain, and HLA-DO mRNA (69). These findings are supported by another study, reporting of CIITA gene-attenuation mediated by Tregs (70). Since Treg cells are a major source of TGFβ, these results are consistence with the earlier observation of MHC-II depletion mediated by Tregs.

#### MHC-II Expression Declines During the Course of Cancer Progression

Treg cells activity (number and function) as well as TGFβ levels increase (in the TME and in the circulation) during the course of cancer development. Based on the findings aforementioned, MHC-II expression is expected to decline during the course of cancer progression. In line with this, MHC-II allele HLA-DRB1*07 expression was higher in lymph nodes of patients with early stage non-small cell lung cancer, compared to patients with an advanced stage of the disease (71).

Taken together, MHC-II expression is important for an effective anti-tumor immunity. Treg-mediated downregulation of MHC-II expression promotes tumor immune escape.

### The Tumorigenic Effect of AIDs Drug-Treatment

#### Non-Specific-Action Immunosuppressants Induce Cancer

Considering the antitumor effects of CD8+ T cells and Th1 cells, it is not surprising that non-specific-action (but not target specific) immunosuppressive drugs increase the risk of cancer. For example, malignancy risk in transplant patients using immunosuppressive drugs such as cyclosporine or azathioprine increases 4 - 500 fold compared to age-matched controls in the general population (72).

## Autoimmune Diseases

### The Involvement of T Cells in Autoimmune Diseases

Autoimmune diseases are a typical example of pro-inflammatory chronic disease (1). Many AIDs display impaired Tregs function (4). Moreover, the lack of Tregs induces AIDs. The immune dysregulation, polyendocrinopathy, enteropathy, X-linked (IPEX) syndrome is a rare recessive disorder caused by mutations in the FoxP3 gene, considered to be the master regulator of Tregs. IPEX is associated with autoimmune enteropathy, Type 1 diabetes (T1D), autoimmune skin disorders and serious infections (73). Since Treg function is impaired in autoimmune diseases, CD4+ T cells activity intensifies. In particular, Th1 and Th17 cells are involved in the pathogenesis of many AIDs (74). Th17 cells frequency or function increases in multiple sclerosis (MS), psoriasis, RA, inflammatory bowel disease, and SLE (75).

Th2 cells mediate allergic immune responses (76) and also play a pathogenic role in systemic sclerosis and ulcerative colitis (74). Systemic frequency of Th2 in RA patients is similar to that in healthy controls (77). The data regarding Th2 cytokines concentration in SLE is conflicting (78, 79).

CD8+ T cells frequency (in affected organs or in the circulation) increases or their function is boosted in MS, systemic sclerosis, T1D, SLE, and severe aplastic anemia (80).

### The Involvement of Neutrophils in Autoimmune Diseases

Neutrophils exhibit a pro-inflammatory function in AIDs. However their modus operandi may vary from one disease to another. The involvement of neutrophils in RA and SLE are described below as representative examples.

Neutrophils in RA patient’s joints are cytotoxic and neutrophils in the circulation of RA patients are primed for the production of ROS, in contrast to those in healthy subjects. Proteases released from lysosomal granules of neutrophils promote RA inflammation (81). Elevated levels of myeloperoxidase (MPO), a cytotoxic enzyme released from neutrophils granules, are found in blood, synovial fluid and tissues of RA patients. MPO increases vascular permeability and allows the penetration of pro-inflammatory immune cells. In addition, MPO attracts more neutrophils to the site of inflammation. Neutrophils in RA synovial fluid secrete inflammatory cytokines such as TNF, B cell-activating factor (BAFF) and receptor activator of nuclear factor kappa B ligand (RANKL). Neutrophil extracellular traps (NETs) are webs of histones and DNA fibers that participate in pathogens eradication. Citrullinated histones comprise around 70% of all NETs proteins. NETs can promote the inflammatory effects of fibroblast-like synoviocytes. NETs formation which is enhanced in RA, is believed to trigger autoimmunity to citrullinated proteins (81).

Similar to their role in RA, neutrophils in SLE display a pro-inflammatory function. They are mainly of the LDN phenotype and secrete type I IFN, IFN-γ, IL-6, IL-8 and TNFα. Like RA, SLE is characterized by enhanced NETs formation. NETs promote immune response in SLE by exposing antigens that are normally shielded by plasma membrane, by reducing T cells activation threshold, and by activation of autoreactive B-cells (82).

### The Involvement of Macrophages in Autoimmune Diseases

In many AIDs macrophages display the M1 pro-inflammatory phenotype (RA, SLE, primary biliary cholangitis, Sjögren’s syndrome, T1D). However, M2 phenotype has been detected in fibrotic AIDs such as systemic sclerosis and inflammatory bowel disease (83). In multiple sclerosis (MS), even though most macrophages in disease lesions display M1 characteristic, a large fraction of macrophages displays both M1 and M2 markers (84).

### The Effect of ROS on Autoimmune Diseases

#### Low ROS Levels Promote Autoimmune Disorders

Chronic granulomatous disease (CGD) is a rare genetic disorder caused by defects in any of the five subunits of the NADPH oxidase complex. An increased frequency of several autoimmune diseases has been reported in CGD patients (85). Experimental evidence (observed in human and animals) supports a link between ROS deficiency and autoimmune diseases such as systemic lupus erythematosus (SLE) (86–88), rheumatoid arthritis (RA) (89–92), psoriasis (93) and Guillain–Barré syndrome (94)

#### Low ROS Levels Impair Treg Function Which in Turn Drive Autoimmunity, While Elevated Levels of ROS Attenuate Autoimmunity

Using a mouse model, Kim et al. have shown that imiquimod-induced psoriatic dermatitis was attenuated by elevated ROS levels, whereas lower ROS levels which induced Tregs dysfunction aggravated the disease (40). It is plausible that impaired Treg function under low ROS levels promoted this autoimmune disease.

#### Very High ROS Levels (Oxidative Stress) Underlie Tregs Damage That Drive SLE and Experimental Autoimmune Encephalitis (EAE)

Strickland et al. demonstrated that oxidant-treated T cells induced lupus-like disease in mice. In addition, genes known to be associated with lupus in SLE patients and animal models of SLE were upregulated by ROS. This effect was most pronounced with peroxynitrite (ONOO-) as the oxidant (95). Peroynitrite is at equilibrium with peroxynitrous acid which rapidly decays in non-alkaline media to •NO_2_ and •OH free radicals (~ 30% yield) and the rest of peroxynitrous acid isomerizes quickly [k = 1.2 sec^-1^ (96)] to the nitrate ion $\left({\text{NO}}_{3}^{-}\right)$ (97). For the treatment of T cells, Strickland et al. have used 20 µM peroynitrite solution (95). This highly oxidative medium triggered the disease.

T cell mitochondrial dysfunction has been proposed as the generator of oxidative stress in SLE (98). Mitochondrial oxidative stress and DNA damage was reported in Treg cells extracted from patients with different AIDs. This mitochondrial oxidative stress and DNA damage which resulted in Treg cell death, was also observed in the EAE mouse model (99). Oxidative stress has been shown to affect the Treg/Th17 balance in SLE (100).

It is evident that oxidative stress (which is synonymous with very high ROS levels) promotes autoimmunity.

#### Immunosuppressive Agents Induce ROS Generation in Renal Transplant Patients

Administration of several immunosuppressive agents to renal transplant recipients (with an uneventful postoperative course and stable renal function) before transplantation, indicated a statistically significant increase in ROS levels (101).

### The Effect of TGFβ on Autoimmune Diseases

#### TGFβ Deficiency Induces Autoimmunity

Blocking TGFβ signaling in T cells induces autoimmunity in mice (102, 103).

### The Involvement of MHC-I in Autoimmune Diseases

#### MHC-I Effect on AIDs Is Ambiguous and Restricted to Few AIDs Only

BXSB-Yaa is a murine-strain that develops spontaneous SLE-like disease. It was demonstrated that β2-microglobulin-deficient BXSB-Yaa mice (BXSB-Yaa mice with a defective MHC-I function) develop more aggressive and lethal SLE-like disease than BXSB-Yaa controls (104). MRL/lpr mouse-strain is another model for spontaneous lupus. β2m-deficient MRL/lpr mice showed accelerated lupus skin lesions accompanied by attenuated nephritis (105). However, other works presented contradicting data: MHC-I deficiency diminished SLE symptoms in a mouse-model of lupus (106) and evoked resistance to experimental induction of lupus in another mouse model (107). The effect of MHC-I on SLE is therefore unclear. It was also reported that MHC-I alleles are associated with only few AIDs (ankylosing spondylitis, psoriasis) whereas MHC-II alleles are associated with more AIDs (RA, T1D, MS, celiac disease and chronic beryllium disease) (108).

It can be concluded that MHC-I is not involved with the pathogenesis of all AIDs and its effect is controversial.

### The Involvement of MHC-II in Autoimmune Diseases

Genetic studies have confirmed that associations between MHC and autoimmune diseases are mostly related to MHC-II alleles. Certain MHC-II alleles increase the probability of developing specific AIDs (a positive association) while other alleles reduce this probability (a negative association). Grave’s disease, narcolepsy, autoimmune thyroiditis, RA, MS, T1D, SLE, ulcerative colitis and Crohn’s disease are all associated with MHC-II polymorphism (109, 110). Ulcerative colitis and Crohn’s disease are associated (but less consistently) with Class I alleles as well (110).

Strong induction of MHC -II expression in mice retina was observed in an experimental mouse-model of autoimmune uveitis (111). Upregulated endothelial MHC-II expression has been reported in dilated cardiomyopathy, RA, SLE, MS and Crohn’s disease patients (112).

Pathogen-stimulated PBMC from subjects homozygous for autoimmune vitiligo high-risk SNP haplotype, demonstrated increased production of IFN-γ and IL-1β than cells from subjects homozygous for a low-risk haplotype (113).

Collectively, AIDs are consistently associated with MHC-II polymorphism. MHC-II expression is upregulated in AIDs and induces a pro-inflammatory effect.

### Cancer Drug-Treatment Drives Autoimmunity

#### Checkpoint Inhibitors Induce Autoimmunity in Patients With a Pre-Existing Autoimmune Disease

Autoimmune disease patients treated with checkpoint inhibitors for cancer are at increased risk of AID relapses. More than 30% of these patients develop flares of their pre-existing AIDs and some develop new autoimmune manifestations (114).

All the aforementioned data concerning cancer and AIDs is summarized in **Table 1** and presented comparatively.

**Table 1:** A comparison of cancer and autoimmune diseases in terms of immunological properties, ROS effect, TGFβ effect and drug effect.

	Cancer	Autoimmune Diseases
pathogenic ROS levels	moderately-high	either low or very high
Innocuous/beneficial ROS levels	either low or very high	moderately-high
MHC-I expression	high (early stage) → low (advanced stage)	low
MHC-I effect on disease	retardation	conflicting data
MHC-II expression	high (early stage) → low (advanced stage)	high
MHC-II effect on disease	retardation	promotion
Treg frequency/function	low (early stage) → high (advanced stage)	low
Treg effect	favorable (early stage) → pathogenic (advanced stage)	favorable
Th1 frequency/function	low	high
Th17 effect	a limited effect	pathogenic
CD8+ T cells activity	high (early stage) → low (advanced stage)	high (in several AIDs)
Neutrophils effect	pro-inflammatory (early stage) → anti-inflammatory (advanced stage)	pro-inflammatory
Macrophages effect	pro-inflammatory(early stage) → anti-inflammatory (advanced stage)	pro-inflammatory
TGFβ effect	antitumor (early stage) → pro-tumor (advanced stage)	TGFβ deficiency induces autoimmunity
Immunosuppressive drugs (untargeted) effect	cancer risk increased	autoimmunity attenuated
Checkpoint inhibitors	improve different advanced cancers	flares in AID patients

## Discussion

### The Comorbidity of Cancer and Autoimmune Diseases

Discussing the comorbidity of cancer and autoimmune diseases, the difference between AIDs developed in the context of pre-existing cancer (paraneoplastic AIDs) and cancer developed in the context of pre-existing AIDs should be acknowledged. Paraneoplastic AIDs are typically alleviated or even resolved by reducing the tumor burden following a surgical removal of the growth or by the use of anti-cancer drugs (115). Paraneoplastic AIDs are generally rare. For example, paraneoplastic neurological syndromes affect one in every 334 patients with cancer (116). There are case reports describing paraneoplastic AIDs such as paraneoplastic polymyalgia rheumatica associated with esophageal carcinoma (117), and paraneoplastic SLE associated with colorectal cancer (118). As an exception, paraneoplastic AIDs associated with lymphoma are not rare and display a 7.6% incidence rate of AIDs in non-Hodgkin’s lymphoma (NHL) and 8.6% in Hodgkin’s lymphoma (HL) patients. The primary AIDs associated with lymphoma are Sjogren’s syndrome in NHL and autoimmune thyroiditis in HL. In most cases, autoimmunity develops after the diagnosis of HL but before the diagnosis of NHL (119).

Paraneoplastic AIDs are often a result of genetic alterations in tumor cells. For example, gene gain-of-function was demonstrated in paraneoplastic cerebellar degenerations with anti-Yo antibodies (Yo-PCD) (rare syndromes caused by an auto-immune response against neuronal antigens expressed by tumor cells). In Yo-PCD ovarian carcinomas, a high percentage of the tumors were found to harbor at least one genetic alteration of Yo antigens which was not observed in ovarian carcinoma samples extracted from patients without Yo-PCD (120).

Systemic sclerosis is another example where genetic alterations trigger an autoimmune response. It was found that tumors from systemic sclerosis patients concurrently diagnosed with cancer, carry mutations in the polymerase III polypeptide A (POLR3A) gene, and the presence of certain anti-nuclear antibodies (anti-Scl70, anti PM/Scl-100, anti RNA Polymerase III) in systemic sclerosis patients increases the risk of cancer (121). It was also found that in a subgroup of scleroderma patients with anti-RNA polymerase I/III autoantibodies, scleroderma developed within 2 years of cancer diagnosis, and nucleolar RNA polymerase III expression was enhanced in tumors of patients in this subgroup only (122). The authors hypothesized that tumors expressing high concentrations of RNA polymerase III initiate an immune response to these autoantigens which triggers scleroderma. The authors also raised the possibility that in some scleroderma patients without cancer pre-diagnosis who are tested positive for anti-RNA polymerase antibodies, scleroderma-specific (originally anti-tumor) immune response aborted the underlying malignancy (122).

Similar mechanisms are reported in cancer developed in patients with pre-existing autoimmune diseases. If tumor cells express antigens to AID autoantibodies, these antibodies may inhibit the antigen related effects. Autoantibodies binding to an antigen with an anti-tumor effect will promote cancer. For example, anti p155/140 antibodies have been detected in sera of adult patients with cancer-associated polymyositis/dermatomyositis (PM/DM) (123). Anti p155/140 antibodies target the transcriptional intermediary factor 1-γ (TIF1-γ). This transcription factor plays an ambivalent role in cancer. TIF1-γ promotes cancer by inhibiting the anti-tumor p-53 gene and by activating estrogen-dependent genes. Accordingly, it displays a pro-tumor effect in breast cancer. However by inhibiting the retinoic acid pathway, TIF1-γ has been shown to suppress liver cancer in a mouse model [Ref. 123 and references therein]. PM and DM are highly associated with the risk of cancer: the SIR value reported for malignancy in PM/DM (averaged over 20 studies) is 4.07 (3.02-5.12) (124).

Even though genetic alterations associated with cancer may elicit immune response that explains the strong associations of certain cancers with autoimmune diseases, this mechanism is either limited to a small number of AIDs or to a small number of patients. The cancer-modulating effect of autoantibodies is also restricted to a few AIDs.

The present work proposes a mechanism that explains the relationship between cancer and AIDs in most AIDs. It is suggested that in the majority of AIDs, the risks of cancer and of cancer-specific mortality are mainly affected by the temporal change of immune response along the course of cancer development.

**Table 1** reveals that several immune reactions, triggers, cytokines and drug effects associated with *advanced* cancer are reciprocal to those associated with autoimmune diseases. The immune reaction to advanced cancer may be regarded as a “high Treg” and anti-inflammatory response while immune reaction to autoimmune diseases is a “low Treg” and pro-inflammatory response. On the other hand, the immune reaction to *early* cancer (prior to immune evasion) is pro-inflammatory, just like the immune reaction to AIDs. The pro-inflammatory environment that underlies AIDs supports the initiation and growth of early malignancy and increases the risk of cancer in AID patients. Indeed, higher incidence of cancer development is reported in many autoimmune diseases (125). In agreement, tumor infiltrating regulatory T cells (which release high amounts of TGFβ in the TME) improve survival in cancers with a long pre-metastatic periods like lymphoma, but have a negative effect on survival in cancers with an early immune evasion like breast cancer, lung cancer, or aggressive squamous cell carcinoma (2).

**Table 2** presents published values of mean standardized incidence ratios (SIR) for different types of cancer, averaged over a large number of AIDs (including 95% confidence intervals). An increased SIR value in cancer patients with a history of AIDs was reported in lung cancer (126), kidney cancer, bladder cancer, prostate cancer (127), hepatobiliary cancer, primary liver cancer, gallbladder cancer, extrahepatic bile duct cancer (128) and multiple myeloma (129). Similarly, SIR for the development of lymphoma, increased in patients with SLE or RA (133). Cancer SIR (averaged over several types of cancer) increased in SLE, Sjogren’s syndrome, systemic sclerosis, and marginally in rheumatoid arthritis (134).

**Table 2:** Standardized incidence ratios (SIR) and cancer-specific hazard ratios (HR) (averaged over a wide-range of autoimmune diseases) for different types of cancer.

Cancer type	SIR (95% confidence interval)	HR (95% confidence interval)	Reference
Lung cancer	1.33 (1.28–1.38)	1.02 (0.98–1.06)	(126)
Kidney cancer	1.44 (1.36–1.53)	0.88 (0.80–0.97)	(127)
Hepatobiliary cancer	1.64 (1.54–1.73)	1.11 (1.04–1.18)	(128)
Liver cancer (primary)	2.13 (1.96–2.31)	0.98 (0.90–1.07)	(128)
Prostate cancer	1.09 (1.06–1.13)	0.88 (0.83–0.94)	(127)
Bladder cancer	1.21 (1.15–1.27)	0.98 (0.88–1.09)	(127)
Gallbladder cancer	1.26 (1.13–1.40)	1.13 (1.01–1.26)	(128)
Extrahepatic bile duct cancer	1.56 (1.29–1.88)	1.31 (1.08–1.59)	(128)
Multiple myeloma (patients age < 60)	1.45 (1.16-1.80)	0.96 (0.70-1.32)	(129)
Breast cancer	0.94 (0.91–0.97)	0.95 (0.89–1.02)	(130)
Cervical cancer	1.03 (0.94–1.13)	0.91 (0.76–1.09)	(130)
Endometrial cancer	0.85 (0.80–0.91)	1.08 (0.88–1.32)	(130)
Ovarian cancer	0.90 (0.84–0.97)	1.09 (0.99–1.20)	(130)
Upper aerodigestive tract and esophageal cancer	N/A	1.20 (1.07–1.34)	(131)
Stomach cancer	N/A	0.93	(131)
Small intestine cancer	N/A	1.18 (0.96–1.44)	(131)
Colon cancer	N/A	1.04 (0.99–1.09)	(131)
Rectum cancer	N/A	1.08 (1.01–1.17)	(131)
Anus cancer	N/A	1.18	(131)
Melanoma	1.0 (0.9-1.1)	0.9 (0.7-1.2)	(132)

N/A, not available.

Turning to the survival of cancer patients with AIDs, the pro-inflammatory nature of AIDs in advanced cancer is expected to slow down cancer propagation and to correct the negative effect of AIDs in early cancer. Since cancer-related mortality occurs almost always at advanced stages of the disease, the hazard ratio for *cancer-specific* mortality is expected to be lower than the cancer standardized incidence ratio. Inspection of **Table 2** reveal that in 11 out of 14 types of cancer the relation {SIR > HR} (where HR refers to cancer-specific mortality) holds true, and in 9 types of cancer, cancer-specific mortality in patients with AIDs is even lower compared to patients without an autoimmune background (i.e. HR < 1), even though the effect may be statistically non-significant in several cancers. In addition, a decrease in mortality was reported in breast cancer patients where a statistically significant 54% reduced risk of breast cancer mortality was observed in women with Th1 dominant autoimmune diseases (135).

In this respect, the distinction between *cancer-specific* mortality and *overall* mortality is important. Overall mortality is affected by comorbidities other than cancer (mainly by comorbidities related to AIDs) and is higher than cancer-specific mortality. This was recorded, for example, in kidney cancer, bladder cancer and prostate cancer (127). Therefore, the relationship {SIR > HR} may not be valid for the hazard ratio of overall mortality.

It must be delineated that the results presented in **Table 2**, are averages over a wide range of autoimmune diseases. The effect of a specific autoimmune disease may strongly deviate from the average.

It should also be realized that factors other than immune reaction, may affect the survival of cancer patients with AIDs differently from those cancer patients without AIDs.

For instance, cancer patients with formerly diagnosed AIDs are often undertreated with respect to their malignancy. The major reason for this insufficient treatment is “poor initial performance status or frailty” (136). An increased frequency of leukopenia due to the simultaneous use of immunosuppressive and chemotherapeutic drugs in these patients is a possible reason for shorter anti-cancer treatment periods or lower doses of anti-cancer drugs. In line with this, higher cancer-specific hazard ratios were reported in breast, cervical, and endometrial cancer in a subgroup of patients with severe AIDs (patients with at least 3 hospitalizations) compared to the mean values over the entire group (that included patients with less severe autoimmune disease) (130). The subgroup with a more severe disease was possibly undertreated.

If insufficient treatment of cancer patients with AIDs is considered as a confounding factor in mortality hazard assessment, the beneficial effect of AIDs on cancer-specific mortality may be even larger, and the “true” HR values should be lower than the values presented in **Table 2**.

To summarize, the incidence of cancer increases in many autoimmune diseases (digestive tract malignancies are exceptions) while cancer-specific mortality of most solid cancers either decreases or is unaffected by pre-existing autoimmune disease. The inequality {SIR > HR} (where HR refers to cancer-specific mortality) is valid in the overwhelming majority of cancer types in patients with formerly diagnosed AID (including digestive tract cancers).

#### The Effect of Reactive Oxygen Species on Cancer and Autoimmune Diseases

**Figure 1:**
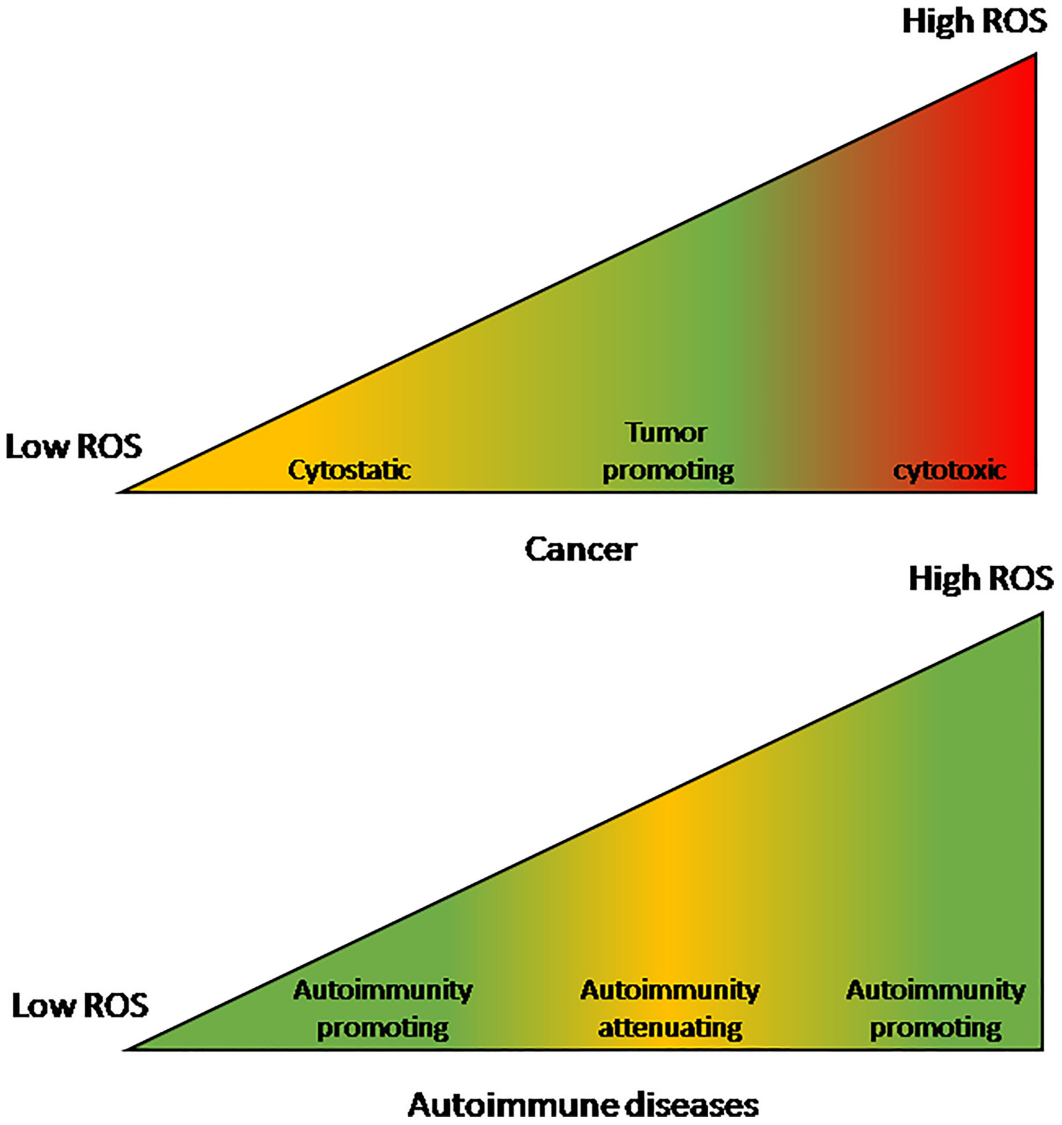
The effect of reactive oxygen species levels on cancers and autoimmune diseases. The effect on cancers is a mirror-image of the effect on autoimmune diseases. Cancers are optimally promoted by moderately-high ROS levels while autoimmune diseases are promoted by either low or very high ROS levels. On the other hand, at moderately-high ROS levels autoimmune diseases are attenuated while very high ROS levels are cancer-cytotoxic and low levels are cancer-cytostatic.

It is noted that ROS levels that promote or suppress cancer are a mirror image of the levels that promote or suppress AIDs (see **Figure 1**). Whereas cancer proliferate and metastasize best under moderately-high ROS levels, AIDs develop and thrive under either low or very high ROS levels. Even though the high-low borders are not well defined, it is clear that very high ROS levels promote AIDs (98). Reactive oxygen species that accumulate in the circulation of AID patients act as pro-cancer agents at early cancer stages but as anti-cancer agents at advanced stages (50). This stage-related effect is another factor that contributes to the pathogenic effect of AIDs in early cancer and to their beneficial effect in advanced cancer, leading to the relationship {SIR > HR} discussed before.

## Summary

Inflammation drives both autoimmune diseases and early stage cancer while it slows down advanced cancer. Low or very high levels of reactive oxygen species induce autoimmunity while cancer propagates optimally at moderately-high levels of reactive oxygen species.

Due to both effects, initiation of cancer and their *early* growth are promoted by pre-existing autoimmune diseases leading to an increased risk of cancer, while *advanced* cancer growth and spread are hindered in patients with pre-existing autoimmune diseases, resulting in an improved survival.

## Author Contributions

The author confirms being the sole contributor of this work and has approved it for publication.

## Author Disclaimer

The views and opinions expressed, and/or conclusions drawn, in this article are those of the author and do not necessarily reflect those of Taro Pharmaceutical Industries Ltd., its affiliates, directors or employees.

## Conflict of Interest

ZE is employed by Taro Pharmaceutical Industries Ltd.

## Publisher’s Note

All claims expressed in this article are solely those of the authors and do not necessarily represent those of their affiliated organizations, or those of the publisher, the editors and the reviewers. Any product that may be evaluated in this article, or claim that may be made by its manufacturer, is not guaranteed or endorsed by the publisher.
